# Assessment of laboratory methods used in the diagnosis of congenital toxoplasmosis after maternal treatment with spiramycin in pregnancy

**DOI:** 10.1186/1471-2334-14-349

**Published:** 2014-06-24

**Authors:** Isolina MX Rodrigues, Tatiane L Costa, Juliana B Avelar, Waldemar N Amaral, Ana M Castro, Mariza M Avelino

**Affiliations:** 1 Laboratory studies of the host-parasite relationship (LAERPH) of Institute for Tropical Pathology and Public Health (IPTSP) of the Federal University of Goiás (UFG), Goiânia, Brazil; 2 Clinical Laboratory of the University Hospital of the Federal University of Goiás (UFG), Goiânia, Brazil; 3 Department of Gynecology and Obstetrics of the Faculty of Medicine - FM/UFG, Goiânia, Brazil; 4 Department of Pediatrics and Puericulture in the Medical School (MS) of Federal University of Goiás (UFG), Av. s/n Setor Leste Universitário, Goiânia-GO CEP: 74001-970, Brazil

**Keywords:** Congenital toxoplasmosis, Treatment, Serology, PCR, Symptoms

## Abstract

**Background:**

The different laboratory methods used in the diagnosis of congenital toxoplasmosis have variable sensitivity and specificity. There is no evidence to prove that maternal treatment reduces the risk of fetal infection. The purpose of this study was to assess methods for the confirmation of congenital toxoplasmosis after maternal treatment with spiramycin during pregnancy, and to evaluate the effect of this treatment on clinical manifestations of the disease in newborns (NB).

**Methods:**

This was a community-based, cross-sectional study of acute toxoplasmosis in newborns at risk of acquiring congenital infection. Participating newborns were born in the Clinical Hospital Maternity Ward of the Federal University of Goiás. Eligible participants were divided into 2 groups: group 1 consisted of 44 newborns born to mothers treated with spiramycin during pregnancy and group 2 consisted of 24 newborns born to mothers not treated with spiramycin during pregnancy because the diagnosis of toxoplasmosis was not performed. The sensitivity and specifity of PCR for *T. gondii* DNA in peripheral blood and serological testing for specific anti-*T. gondii* IgM and IgA, and the effects of maternal spiramycin treatment on these parameters, were determined by associating test results with clinical manifestations of disease.

**Results:**

The sensitivity of the markers (*T. gondii* DNA detected by PCR, and the presence of specific anti-*T. gondii* IgM and IgA) for congenital toxoplasmosis was higher in group 2 than in group 1 (31.6, 68.4, 36.8% and 3.7, 25.9, 11.1% respectively). Even with a low PCR sensitivity, the group 2 results indicate the importance of developing new techniques for the diagnosis of congenital toxoplasmosis in newborns. Within group 1, 70.4% of the infected newborns were asymptomatic and, in group 2, 68.4% showed clinical manifestations of congenital toxoplasmosis.

**Conclusions:**

The higher proportion of infants without clinical symptoms in group 1 (70.4%) suggests the maternal treatment with spiramycin delays fetal infection, reducing the clinical sequelae of the disease in newborns. Given the low sensitivity of the tests used, when there is suspicion of congenital transmission several serological and parasitological tests are required in order to confirm or exclude congenital toxoplasmosis in newborns.

## Background

Since the discovery of *Toxoplasma gondii* (*T.gondii*) by Alfonso Splendore in 1908, many studies have attempted to explain its mechanisms of transmission and the host immune response to the parasite, to discover drugs to inhibit its proliferation, and to develop excellent diagnostic techniques using highly sensitive and specific immune and bio-molecular methods [1-6]. The rates of transmission and clinical manifestation of congenital toxoplasmosis vary sharply in newborns (NB, and depend on the immune response of the fetus to the parasite, the gestational age when infection took place, the parasite load in the fetal circulation at the time of infection, the genotype of the parasite, and the specific treatment administered to the pregnant patient [1,2]. Clinical follow-up of cases of suspected congenital transmission is very difficult once the pregnant woman has been treated with spiramycin, which affects the identification of the parasite in the NB [1,2]. In addition, tests for anti-*T.gondii* IgM and IgA have poor sensitivity in fetuses and NB [6-9]. Most infected NB (60%) are asymptomatic [1,2]; however, they may develop severe sequelae such as blindness or mental retardation if not treated [1,2,10-12].

It has not yet been conclusively proven that maternal treatment reduces the risk of fetal infection [3,13-19], although some studies have demonstrated a beneficial effect of treatment [20-27]. Moreover, treatment with a combination of pyrimethamine and sulphadiazine is thought to be more effective than spiramycin treatment for reducing the risk of clinical manifestations in infected children [1,2,20-27]. There are conflicting views in the literature regarding the effectiveness of spiramycin treatment when the fetus has already been infected [1,2]. However, many authors believe that spiramycin is able to reduce the severity of fetal infection by delaying the onset of fetal disease and thus allowing greater maturation of the fetal immune system [28-30].

A number of studies have evaluated the performance of polymerase chain reaction (PCR) using amniotic fluid samples for the diagnosis of fetal infection [31-35]. In addition, in some countries cerebrospinal fluid (CSF) is the most frequently used diagnostic sample [1], while in others placental samples are used [36]. However, few studies have attempted to evaluate the sensitivity of PCR testing of the peripheral blood of NB congenitally infected with *T.gondii*[37]. Considering the difficulty of diagnosing congenital toxoplasmosis and the low frequency of clinical manifestations in infected NB and infants, the purpose of this study was to evaluate the influence of prenatal spiramycin treatment on the sensitivity and specificity of diagnostic tests, and its effects on clinical manifestations of the disease in NB.

## Methods

### Population

This was a community-based, cross-sectional study of acute toxoplasmosis in NB considered at risk for acquiring congenital infection; all NB participants were born in the Maternity Ward of the Clinical Hospital (HC) of the University Federal of Goiás (UFG) (Brazil) between October 2004 and December 2011. All NBs were selected by the Maternity Protection Service of the state of Goiás and by the Congenital Infection Control Program in the Maternity Ward at HC/UFG based on their mothers having tested positive for specific anti-*T.gondii* IgM and IgG during the prenatal or postpartum periods.

Toxoplasmosis testing was performed at the first prenatal care visit during the first gestational trimester. The pregnant patients were considered to have an acute infection when presence of *T. gondii*-specific IgM antibodies were confirmed and when the avidity of IgG was low (<30%), as described by Jenum et al. [38]. All tests were performed before 16 weeks of pregnancy. Treatment of acutely infected pregnant women began as soon as the diagnosis was delivered to the physician (15 days after the test). The state program does not provide serological follow-up of seronegative pregnant women, whereas the Maternity Ward at HC routinely conducts thorough toxoplasmosis testing of all NB and their mothers. This postpartum testing detects children with likely congenital infection, but does not indicate the time when the acute infection occurred during the pregnancy. All suspected NB were closely followed for one year, while a longitudinal study with monthly clinical and laboratory evaluations was being conducted. These examinations routinely consisted of laboratory tests for toxoplasma-specific IgG and IgM antibodies, mouse inoculation for parasitological diagnosis of congenital toxoplasmosis, ocular fundic examinations by indirect ophthalmoscopy, and transfontanellar ultrasonography of all infants at risk of congenital infection. In addition to the detection of IgG and IgM, testing for toxoplasma-specific IgA antibodies and PCR of peripheral blood samples for identification of *T. gondii* DNA were added to these examinations as part of the study protocol.

There were 247 children born with suspected congenital toxoplasmosis during the study period. The patients were either the children of women with suspected acute infection during pregnancy or children whose mothers had seroconverted (women previously identified as seronegative who did not undergo seroconversion surveillance during pregnancy). Of the 99 patients who underwent the diagnostic testing required by the study protocol (PCR and testing for toxoplasma-specific IgA, IgM, IgG), 31 NB were excluded because the diagnosis of toxoplasmosis was not completed due to lack of clinical and laboratory monitoring. The tests were performed during the neonatal period and the patients underwent clinical and laboratory follow-up for at least one year.

**Figure 1:**
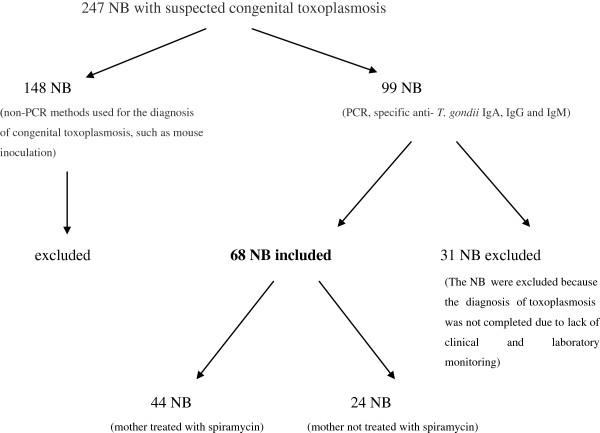
Flowchart.

From interviews with the mothers, the presence or the absence of specific treatment with spiramycin during pregnancy was investigated. Forty-four of the mothers had been treated with 3 g of spiramycin daily, administered over 3 doses per day (i.e. 3 × 1 g), from the time of acute toxoplasmosis diagnosis until birth. Twenty-four mothers had not been treated because toxoplasmosis testing was not performed during pregnancy (these mothers served as the control group for the treated ones). The NB were divided into two groups: group 1–44 NB born to mothers treated with spiramycin during pregnancy; and group 2–24 newborns born to mothers who did not receive spiramycin treatment during pregnancy.The study was approved by the Human and Animal Experimentation Ethics Committee of the HC/UFG (protocol n. 092/2001 and protocol n. 024/2010) and the mothers of the NB who agreed to participate in the study gave their informed consent after they had been informed of the importance of the research Figure 1.

### Criteria for the diagnosis of congenital toxoplasmosis

The Program for Congenital Infection Control at the HC Maternity Ward of UFG performs clinical follow-up from birth until at least 12 months of age of all infected NB and those with suspected congenital toxoplasmosis. A patient is considered infected when:

•*T. gondii* is detected in peripheral blood or CSF by mouse inoculation or *T. gondii* DNA is detected by PCR;

•specific anti-*T.gondii* IgA and/or IgM is identified in fetal or NB blood;

•specific antibodies (IgG and/or IgM) are found in the CSF of the NB;

•fetal or NB *T. gondii*-specific IgG is 4X greater than maternal IgG;

•NB specific anti-*T. gondii* IgG levels increase, or remain positive after 12 months of life;

•the patient has clinical symptoms compatible with congenital toxoplasmosis infection and not explained by another diagnosis (such as Chagas disease, syphilis, rubella, cytomegalovirus, HIV, HTLV, hepatitis B and C), and the presence of IgG anti *T. gondii* until 12 months of children's lives.

The presence of specific anti-*T. gondii* IgM and IgA was confirmed using a new blood sample collected between the fifth and tenth day of life.

### Biological techniques used for the diagnosis of congenital toxoplasmosis in the neonate

Detection of specific anti-*T.gondii* IgM and IgA antibodies during serological testing, or detection of *T.gondii* DNA in peripheral blood by PCR were considered to indicate congenital *T.gondii* infection.

#### Immunoassay

Specific anti-*T.gondii* antibodies were detected using several assay systems according to the manufacturer's instructions. Toxoplasma-specific IgG and IgM were detected by microparticle enzyme immunoassay (MEIA) performed using an AXsYM immunoassay system (Abbott Laboratories) and an Architect i4000 (Abbott Laboratories) with Chemi-Flex technology. IgM was also detected by enzyme-linked fluorescent assay (ELFA) performed using a VIDAS immunoanalyzer (Biomérieux). IgA was detected using an antibody-capture enzyme immunoassay.

#### PCR

Peripheral blood was collected from the NB before treatment with sulfadiazine, pyrimethamine, and folinic acid commenced. A Pure Link Genomic Purification kit (Invitrogen) was used to extract the DNA according to the manufacturer’s specifications. The PCR reactions were performed in a MasterCycler Personal thermocycler. The amplification process consisted of an initial denaturation at 94°C (5 min), 35 cycles of denaturation at 94°C (1 min), annealing at 62°C (1 min) and extension at 72°C (1 min), followed by a final extension at 72°C (10 min). The PCR reactions were performed in duplicate, using a sequence of the B1 gene of *T.gondii* as the target sequence. The following primers were used: Toxo-B5 (5’-TGA AGA GAG GAA ACA GGT GGT CG-3’) and Toxo-B6 (5’-CCG CCT CCT TCG TCC GTC GTA-3’). The PCR products were separated by electrophoresis in a 6% polyacrylamide gel and visualized after staining with silver nitrate [39]. Peritoneal fluid from mice infected with the RH strain of *T. gondii* was used as a positive control.

### Statistical analysis

The data were entered into Microsoft Excel 2007 and univariate analysis was conducted using Epi Info version 3.3.2. The associations between each variable collected were tested and P values < 0.05 were considered statistically significant, using confidence intervals of 95%. The Fisher's Exact Test was performed when the frequency was less than 5.

A standardized form was used for data collection. The information collected included: maternal data (type of treatment, when the diagnostic testing was performed and the instructions given by the physician to seronegative patients regarding prophylactic measures); NB clinical data (signs of a possible congenital infection); and NB laboratory data (detection of *T.gondii* DNA by PCR analysis or specific anti-*T.gondii* IgM and IgA antibodies).

The sensitivity and specificity of the laboratory tests were determined for both groups of infants. Statistical analysis was performed in order to verify the existence of an association between laboratory markers of congenital infection with maternal treatment and with the presence or absence of clinical manifestations of the disease in the NB.

## Results

Congenital toxoplasmosis was confirmed in 61.4% (27/44) of the NB in group 1 and 79.2% (19/24) of the NB in group 2. Women from group 1 had received prenatal treatment for toxoplasmosis and almost all of these women had undergone serological testing during the first trimester because of suspicion of acute toxoplasmosis. The women from group 2 who had been seronegative for toxoplasmosis during first trimester screening had probably seroconverted during the 2nd or 3rd trimester. Of the mothers in group 2 who gave birth to infected NB, 89.5% (17/19) had been seronegative for *T. gondii* during the first 3 months of pregnancy and 10.5% (2/19) had not undergone prenatal toxoplasmosis testing. None of these seronegative pregnant women were screened for seroconversion during pregnancy; and the patients reported that they had not been informed of prophylactic measures to prevent infection by *T.gondii*.

Laboratory markers of congenital infection (*T.gondii* DNA or *T. gondii*-specific IgM and IgA antibodies) were more frequently detected in infected NB from group 2. However, treatment with spiramycin did not interfere significantly with laboratory detection of congenital *T.gondii* infection (Table 1). The sensitivity of the laboratory tests was higher for infants in group 2, while the specificity was similar for both groups (100%).

**Table 1:** Positivity of biological markers of congenital toxoplasmosis in infected newborns from mothers treated with spiramycin (Group 1) and from untreated mothers (Group 2)

	**Group 1**		**Group 2**		**p**^**a**^
	**Infected (N = 27)**		**Infected (N = 19)**		
	**n% Sensibility (%)**		**n% Sensibility (%)**		
**PCR**	1 (3,70)	3,7	6 (31,58)	31,58	0,053
**Positive**					
**Negative**	26 (96,30)		13 (68,42)		
**IgM**	7 (25,93)	25,93	13 (68,42)	68,42	0,0662
**Positive**					
**Negative**	20 (74,07)		6 (31,58)		
**IgA**	3 (11,11)	11,11	7 (36,84)	36,84	0,0969
**Positive**					
**Negative**	24 (88,89)		12 (63,16)		

^a^Fisher’s Exact Test.

(Goiânia-GO, Brazil, 2012).

Examination of infected NB revealed that clinical manifestations of congenital toxoplasmosis occurred in 29.6% of infants in group 1 and 68.4% of infants in group 2 (Table 2). Treatment of pregnant women with spiramycin significantly decreased the occurrence of hydrocephalus, neurological impairment, and retinal lesions.

**Table 2:** Frequency of clinical manifestations of congenital toxoplasmosis in newborns from mothers treated with spiramycin (Group 1) and from untreated mothers (Group 2)

**Any of the following clinical signs**	**Group 1**	**Group 2**	**p**^**a**^
	**(N = 27)**	**(N = 19)**	
	**n%**	**n%**	
Presence	8 (29,63)	13 (68,42)	0,0963
Cerebral calcification	1 (3,70)	3 (15,79)	0,2190
Hydrocephalus	-	5 (26,32)	0,0181
Microcephaly	-	2 (10,53)	0,1862
Blindness	-	2 (10,53)	0,1862
Chorioretinitis	1 (3,70)	3 (15,79)	0,2190
Corticosubcortical dysfunction	2 (7,40)	3 (15,79)	0,3677
Systemic toxoplasmosis	-	1 (5,26)	0,4255
Optic neuropathy	-	4 (21,05)	0,0384
Hepatosplenomegaly	-	2 (10,53)	0,1862
Hearing dysfunction	4 (14,80)	1 (5,26)	0,3402
Generalized lymphoglandular form	1 (3,70)	-	0,5957

^a^Fisher’s Exact Test.

(Goiânia-GO, Brazil, 2012).

The presence of serological markers for toxoplasmosis was not associated with greater severity of congenital infection.

## Discussion

In Goiânia (2003), Avelino et al. [40] found one of the highest rates of toxoplasmosis seroconversion during pregnancy described in the literature (8.6%). This result led to the introduction of a public program to control toxoplasmosis during pregnancy in the state of Goiás. Although the treatment of toxoplasmosis in pregnancy is not followed by a reduction in its transmission to the fetus [13-16], it has been shown that treatment can reduce the severity of fetal infection [17-27] as we found in this study. However, in our study as in others [11,14,18], some children born to treated mothers developed ocular or neurological sequelae to congenital infection. However, severe clinical manifestations of congenital infection were found only among children born to untreated women (Table 2).

Our results highlight flaws in the implementation of primary prophylactic measures for seronegative women at risk of becoming infected by *T. gondii*. Of the mothers in group 2, 89.5% gave birth to infected children. Moreover, they were not informed of possible prophylactic measures. In France, implementation of a prevention program and screening of pregnant women led to a reduction of symptomatic neonates [24]. Preventive measures should be taken throughout prenatal care (by doctors or the healthcare professional team). Additional information on how to prevent the occurrence of acute infection during pregnancy should be provided. Simple measures such as avoiding undercooked meat, eggs and raw vegetables; not drinking untreated water or unpasteurized milk; using hygienic gardening practices; and limiting interaction with cats can help to prevent infection [41]. Failure to provide information on primary prophylaxis measures, and the lack of seroconversion surveillance during prenatal care, were responsible for the high incidence of congenital infection among NB born to the initially seronegative pregnant women of group 2. There is a high possibility of contact between pregnant women and the sources of the parasite in our environment [40]. In addition to failure of primary prophylaxis, the initially seronegative women in this study did not undergo the seroconversion surveillance by monthly serologic screening that is indicated in regions with a high prevalence of toxoplasmosis [1,2,42,43]. Thus, many women were acutely infected but not properly diagnosed and treated.

Of the children in group 2 (untreated mothers), 68.4% (13/19) were born with severe clinical conditions (Table 2). In contrast, only 29.6% of the NB in group 1 were symptomatic and none had severe clinical disease. Thirteen children required tertiary prophylactic measures as their mothers had been untreated. They needed to be followed by a multidisciplinary team and the cost was estimated to be exorbitant for the government program. According to Remington et al. [1], such costs could reach a million US dollars during the lifetime of a patient. This could have been alleviated if those women had followed prophylactic measures against *T.gondii* and had also undergone seroconversion surveillance. Only 10.5% (2/19) of the untreated women had not undergone prenatal care.

The higher percentage of infants without clinical symptoms in group 1 (70.3%) compared to group 2 (31.5%) suggests that maternal treatment with spiramycin reduced the fetal load of *T.gondii*, thus minimizing the sequelae of congenital toxoplasmosis infection [28-30]. Research conducted by Andrade et al. [44] showed that 60% of infants born to treated mothers were asymptomatic, which is similar to our results. However, NB outcomes after maternal spiramycin treatment are variable within the literature, with results ranging from no benefit [13-15] to reduced severity of congenital infection [17-27]. A European multicenter study [14] concluded that spiramycin was of no benefit in reducing the severity of fetal infection. However, recent studies have shown that early treatment can interfere with the transmission of infection, and decrease the severity of congenitally acquired toxoplasmosis [23-25]. A review by Wallon et al. [3] reported conflicting results within the literature; five studies showed that maternal treatment reduced the severity of vertical transmission, but this was not confirmed in another four studies. Still other studies, such as that by Foulon et al. [13], have shown that early treatment was able to reduce the severity of the clinical consequences of congenital infection, but not vertical transmission to the fetus. Hohlfeld et al. [12] demonstrated that treatment slightly reduced the severity of congenital infection.

For the infected children whose mothers were not treated it was not possible to determine when maternal infection took place. Severe neurological lesions were observed only among the children of untreated mothers, suggesting that maternal treatment contributed to a decrease in the development of severe neurological lesions. The same result was shown by the European Multicenter Study [45]. However, Cortina-Borja et al. [46], found no reduction in the severe neurological sequelae affecting children of acutely infected mothers, regardless of whether maternal treatment consisted of spiramycin or the combination of pyrimethamine and sulfadiazine. Those authors report that 31% of the children born to mothers acutely infected by *T. gondii* during pregnancy had severe neurological sequelae. This is a higher percentage than reported in regions that do not have preventive government programs, indicating a more aggressive strain of toxoplasmosis circulating in this environment [46].

The severity of the infection was greatest in NB from group 2; 68.4% were symptomatic at birth and developed severe manifestations of congenital toxoplasmosis: 4 (30.8%) had neuro-optical lesions, 2 (15.4%) blindness, 5 (38.5%) hydrocephalus, 2 (15.4%) microcephaly, and 1 (7.7%) systemic toxoplasmosis. Gilbert et al. [26], showed that the risk of being born with clinical signs of congenital infection, or developing signs until the age of three years, is lower in countries in which intensive treatment of pregnant women is performed, such as in Austria.

Olariu et al. [47] reported a clinical picture as severe as that found in Goiania for untreated children. One or more severe clinical manifestations of congenital toxoplasmosis were reported in 84% of the affected infants. These manifestations included eye lesions (92.2%), brain calcifications (79.6%), and hydrocephalus (67.7%). In 61.6% of the infants, eye lesions, brain calcifications, and hydrocephalus were present simultaneously. Soares et al. [48] found that 72.4% of patients appeared asymptomatic at birth, but 34.5% had chorioretinitis, 32.3% had intracranial calcification, and 42.9% had neuromotor delay.

Hearing dysfunction was observed in five NB in our study, 4 (14.8%) from group 1 and one (5.3%) from group 2 (Table 2). Andrade et al. [44] found auditory dysfunction in six children with congenital toxoplasmosis, two of whom were born to mothers who were properly treated for toxoplasmosis during pregnancy. These data suggest that congenital toxoplasmosis, even in the absence of other clinical manifestations, should be considered in the evaluation of children with hearing loss, and that maternal treatment does not exclude the possibility of developing this dysfunction. Brown et al. [49] reported hearing impairment in 28% of children born to untreated mothers; however, in our study there was a higher incidence of hearing loss in the treated group.

The sensitivity of serological diagnostic markers of congenital toxoplasmosis in NB (*T. gondii*-specific IgM and IgA antibodies and PCR evidence of *T. gondii* DNA) reported in the literature is low, with independent studies reporting different results (1,2,6–9,32–36). Diagnosis seems to be adversely affected by maternal treatment as reported by Gilbert et al. [9]. In our study, *Toxoplasma gondii* was not detected by PCR in 96.3% (26/27) of samples from NB born to treated mothers, indicating that spiramycin treatment reduced the sensitivity of this technique. A similar phenomenon was reported by Rodrigues et al. [50], Fricker Hidalgo et al. [51] and Bessières et al. [8]. There was also low PCR sensitivity (31.6%) in group 2 NB, suggesting that peripheral blood may not be the best biological sample to use for the diagnosis of congenital toxoplasmosis. Sterkers et al. found an even lower percentage (21.2%) of PCR-positive congenitally infected NB in 2012 [31], and in 2001 Bessières et al. reported levels similar to those we found in this study [8]. The identification of the parasite in the amniotic fluid is easier than its detection in the NB, thus the sensitivity of the diagnostic technique (either PCR or mouse inoculation) is greater when there is intrauterine parasitemia. Spalding et al. [52] suggest that the low sensitivity of PCR for the diagnosis of NB with congenital *T. gondii* infection relative to its high sensitivity when used to test amniotic fluid is due to the transient persistence of *T.gondii* in peripheral blood [9,33-35]. However, according to Okay et al. [53], the amniotic fluid from only 40.47% of congenitally infected NB was PCR-positive [53]. However, despite these difficulties, PCR can be highly sensitivity (around 90%) if it is used to test amniotic fluid collected close to the time that the pregnant woman seroconverted, which is the time of acute fetal infection [1,2,9,32-35]. Bessières et al. [8] found a PCR sensitivity of 43% when using umbilical cord blood, which is higher than the sensitivity that we report for peripheral blood.

The sensitivity of specific anti-*T.gondii* IgM and IgA antibodies in NB of both groups (43.5 and 21.7%, respectively) was similar to that observed in other studies. Pinon et al. [7] found the sensitivity of anti-*T.gondii* IgM and IgA in NB samples to be approximately 25%. Naessens et al. [54] reported a sensitivity of 40% for IgM. Bessières et al. [8] found greater sensitivities for IgA (60%) and IgM levels (50%) than those found in this study. Foulon et al. [13] found positive results for IgA (58%) and IgM (54%) using the enzyme-linked immunosorbent assay (ISAGA). In a study involving 14 laboratories supported by the European Community Biomed 2 program, Pinon et al. [55] evaluated immunologic methods for the postnatal diagnosis of congenital toxoplasmosis and compared ELFA with a commercial enzyme immunoassay (EIA) or in-house immunosorbent agglutination assay (ISAGA) for the detection of IgM or IgA. The results were highly sensitive when the techniques were combined.

Treatment of pregnant women with spiramycin did not interfere, from a statistical viewpoint, with the sensitivity of specific anti-*T.gondii* IgM and IgA antibodies in NB within the 2 groups (Table 1). This is similar to the findings of other studies [8]. However, the sensitivity was higher (68.4% for IgM and 36.8% for IgA) in infants from group 2, similar to the findings of Lebech et al. [56]. It is likely that maternal treatment reduced the parasite load transmitted to the fetus, consequently reducing antigenic stimulation and the fetal humoral immune response. This, along with delayed fetal disease, may be one of the reasons for the reduced incidence of neuro-optical lesions and hydrocephalus in babies born to treated mothers.

Detection of specific anti-*T.gondii* IgA did not improve early diagnosis of congenital toxoplasmosis in the study group, because in 90% (9/10) of the infants with anti-*T. gondii* IgA, IgM was also present. However, IgA seemed to be the marker with the poorest prognosis for the congenital infection, as it was present in all NB who developed the neuro-optical form of toxoplasmosis (8.7% or 4/46). The presence of other serological markers for toxoplasmosis was not associated with greater severity of congenital infection.

The high specificity of the laboratory markers for congenital toxoplasmosis (PCR, specific IgM and IgA) observed in this study reinforces the need for such procedures to be performed in the routine diagnosis of congenital infection. They add parameters to confirm the diagnosis and need for subsequent treatment, which uses toxic drugs in patients suspected of congenital infection.

The effectiveness of treating pregnant women in order to prevent fetal infection is highly controversial [12-27]; however, our results suggest that treatment of the mother reduces the severity of fetal infection. The poor prognosis observed in infected group 2 NB suggests that the guidelines of the Program for Congenital Infection Control from France [57,58] should be followed, including monthly serologic testing throughout pregnancy for seronegative patients and a focus on earlier medical treatment using sulfadiazine, pyrimethamine and folinic acid instead of spiramycin [1,2,12,18-27] in case of proven fetal infection. Earlier diagnosis and treatment may prevent vertical transmission and the treatment of pregnant women identified as having recent toxoplasmosis can reduce the severity of the fetal infection [24,25].

### Limitations of the study

Randomized controlled trials on pregnant women, which could provide higher statistical weight to this research, were not performed due to ethic reasons. Not with standing, the scientific knowledge still holds very conflicting opinions toward the effectiveness of various treatments. We hope this work demonstrates that spiramicin can also be useful in reducing the seriousness of the fetal infection, as previously indicated by previous work that have been recently very criticized.

## Conclusions

Spiramycin treatment of women with acute toxoplasmosis did not interfere significantly with the detection of laboratory markers of congenital toxoplasmosis. Treatment did reduce the frequency of the clinical manifestations in the NB, minimizing the severity of the congenital infection.

The detection of laboratory markers for congenital infection was positively correlated with the clinical diagnosis of toxoplasmosis in NB from both groups. However, due to the low sensitivity of the laboratory markers, negative results do not exclude the possibility of congenital infection. These results emphasize the importance of clinical follow-up of NB suspected to have congenital toxoplasmosis until at least 12 months of age.

## Abbreviations

NB: Newborn; T. gond: Toxoplasma gondii; IgG: Immunoglobulin G; IgM: Immunoglobulin M; IgA: Immunoglobulin A; HC: Clinical hospital; UFG: Federal University of Goiás; CSF: Cerebrospinal fluid; PCR: Polymerase chain reaction; HIV: Human immunodeficiency virus; HTLV: Human cell lymphotropic virus; LAERPH: Laboratory studies of the host-parasite relationship; IPTSP: Institute of Tropical Pathology and Public Health; MEIA: Microparticle enzyme immunoassay; ELFA: Enzyme-linked fluorescent assay; ELISA: Enzyme-linked immunosorbent assay; ISAGA: Immunosorbent agglutination assay.

## Competing interests

The authors declare that they have no competing interests.

## Authors’ contributions

All authors contributed to the design of the study, prepared and approved the final manuscript. IMXR – responsible for conducting laboratory tests of the umbilical cord blood, NB suspected of congenital infection (beyond the 5th day of life) and cerebrospinal fluid, performed in the Laboratory of Immunology HC/UFG and also for laboratory monitoring of patients suspected of congenital infection; TLC – carried out the immunoassays, identification of *Toxoplasma gondii* by PCR and participated in the writing of the manuscript; JBA - responsible for the *T. gondii* isolation, serology of special fluids - WNA – responsible for collecting the biological material from the fetus and the mother with acute toxoplasmosis and for the treatment of the acutely infected pregnant woman; AMC – responsible for identification of *Toxoplasma gondii* by PCR and inoculacion in mice, participated in the project since its implementation and also responsible for the writing and analysis of the study data. MMA – Corresponding author, responsible for the toxoplasmosis project in Goiânia, the collection of results, data analysis, monitoring children suspected of congenital infection and writing the manuscript.

## Level of interest

This article in the field of immunology shows the effectiveness of maternal treatment in reducing the clinical manifestations of congenital toxoplasmosis.

## Pre-publication history

The pre-publication history for this paper can be accessed here:

http://www.biomedcentral.com/1471-2334/14/349/prepub
